# Bipolar hemiarthroplasty for femoral neck fractures in elderly patients: a retrospective study of 1001 patients

**DOI:** 10.1007/s00402-025-06073-7

**Published:** 2025-10-13

**Authors:** Filippo Gerber, Daniel Wagner, Geneviève Perrenoud, Matthaios Papadimitriou-Olivgeris, Sylvain Steinmetz

**Affiliations:** 1 Department of Orthopedics and Traumatology, University Hospital of Lausanne, Lausanne, Switzerland; 2 University of Lausanne, Lausanne, Switzerland; 3 Infectious Diseases Service, University Hospital of Lausanne, Lausanne, Switzerland

**Keywords:** Femoral neck fractures, Geriatric orthopedics, Acetabular erosion, Prosthetic joint infection, Dislocation, Bipolar hemiarthroplasty

## Abstract

**Introduction:**

Displaced intracapsular femoral neck fractures (FNF) are the most frequent surgical pathology in orthopedics. Controversy surrounds optimal treatment, with little consensus, particularly in the elderly multimorbid at risk population. For over a decade, our institution adopts a standardized protocol for FNF, utilizing cemented hip hemiarthroplasty (HA) via the posterior approach. This study evaluates the outcomes of this approach, contributing to the ongoing debate and potentially guiding future treatment strategies.

**Methodology:**

This retrospective study included patients (≥ 60 years) who underwent HA for FNF from January 1, 2008, to June 30, 2019, at a University Hospital. Our primary endpoint was revision surgery for HA (rHA) within four years after HA. Secondary endpoints included hip-related and unrelated complications.

**Results:**

Of 1001 patients, 40 (3.9%) underwent rHA. Indications were periprosthetic fractures (5; 0.5%), dislocation (15; 1.5%), suspected prosthetic joint infection (17; 1.7%), and acetabular erosion (3; 0.3%). Four-year mortality was 51%. Cox regression revealed age > 80 years (aHR 1.86, *p* < 0.001), ASA score III or IV (aHR 2.11, *p* < 0.001), and postoperative delirium (aHR 1.29, *p* < 0.05), as independent predictors of higher 4-year mortality. No difference was observed among patients with and without revision indication for ASA scores III or IV (64% vs. 64%; *p* = 0.912), surgery within 24 h (52% vs. 57%; *p* = 0.334), duration over 90 min (46% vs. 53%; *p* = 0.201), and surgery during the night shift (24% vs. 19%; *p* = 0.285). Board certification did not impact revision rates (39% vs. 39%; *p* = 1.000).

**Conclusion:**

Cemented HA is a safe and reliable treatment option for FNF, delivering consistent outcomes in the elderly multimorbid population, with low rates of rHA (3.9%). The posterior surgical approach, even in patients with heightened dislocation risk, remains a viable option. Timing of surgery (night/day) does not significantly affect revision rates, which could have substantial implications for surgical planning and healthcare resource allocation.

## Introduction

Displaced intracapsular femoral neck fractures (FNF) represent one of the most common surgical challenges encountered in contemporary orthopedic departments globally, with 1.8 million cases occurring annually, and projected to exceed 5 million in 2050 [1]. Data from the Swiss National Implant Registry (SIRIS) reflects this trend with an annual increase of 7–8% per year [2]. The economic burden is substantial, with health and societal costs at one year postoperatively estimated as high as USD 43,000 per patient [3]. This will increasingly strain healthcare resources and funding, necessitating more efficient management strategies [4, 5].

Debates in the management of FNF include: osteosynthesis or replacement in non-displaced fractures; hemiarthroplasty (HA) versus total hip arthroplasty (THA) in displaced fractures, and whether to employ cemented or non-cemented stems [6–9]. There is some evidence that THA may reduce the risk of reoperation, although the optimal implant choice remains controversial [6].

HA is generally recommended for patients aged 70 years and older without osteoarthritis [8, 10]. In contrast, THA is often preferred for more active, and cognitively intact individuals, due to a superior long term functional outcome [11, 12]. However, the functional benefits of THA are not consistently observed in populations >80 years [13]. Often, studies which report superiority of THA employ exclusion criteria omitting cognitively impaired patients and octa-nonagenarians [14–16]. These sub-populations constitute a large proportion of patients presenting with FNF.

The advantages of HA in the geriatric population are shorter surgical time and lower dislocation rate [5, 17–20]. A meta-analysis demonstrated comparable patient survivorship for HA and THA in elderly patients, with HA showing fewer dislocations and THA not presenting the risk of acetabular erosion [21]. Despite that, a trend towards THA for FNF has emerged. SIRIS data indicate an increased rate of FNF treated with THA from 38.5% in 2017 to 47.3% in 2022, while age distribution remained constant [2].

Cemented HA is widely accepted for treating FNF in the geriatric population particularly as these patients present with significant comorbidities and a limited life expectancy with a one-year mortality of 30% [13]. Postoperative complications including periprosthetic fractures (PPF), periprosthetic joint infections (PJI), dislocations, and acetabular erosion, are common (up to 16%) and contribute to varying revision surgery rates [11, 22–24]. Identifying risk factors for these complications is crucial to improving patient outcomes, reducing mortality, and controlling rising healthcare costs.

In the absence of clear treatment recommendations, various guidelines are adopted [1, 25]. We treated elderly patients with displaced FNF with cemented HA. The primary aim of this study is to analyze revision rates in patients over the age of 60 treated with HA for FNF. The secondary aim was to identify independent non-hip-related risk factors associated with revision rates and to describe hip-related complications. We hypothesize that HA is an appropriate choice of management for the treatment of FNF in the elderly.

## Methods

### Study design and setting

This retrospective monocentric cohort study includes patients treated at the Department of Orthopedics and Traumatology at Lausanne University Hospital in Switzerland between January 1 st 2008 and June 30th 2019 (11.5 years) for a displaced FNF. The manuscript was written in accordance with the STROBE (Strengthening the Reporting of Observational Studies in Epidemiology) guidelines. The primary endpoint was surgical revision (ORIF for periprosthetic fracture, component exchange, debridement with component exchange, implant removal or conversion to THA) of HA within a minimum of 4 years following HA. Secondary outcomes included hip related and non-hip related complications. The study was approved by the Human Research Ethics Committee of the Canton of Vaud, Switzerland (CER-VD 2018 − 01456).

### Participants

Inclusion criteria were elderly patients (≥ 60 years) with a displaced intracapsular FNF (Garden III or IV) requiring surgical treatment by HA or revision of failed treatment in non-displaced FNF. Exclusion criteria were refusal to consent, pathological or basicervical fractures.

### Data collection and definitions

All patient data were extracted via our institution’s electronic medical health record software (Soarian - Cerner, North Kansas City, MI, USA) and surgical records via our institution’s surgical database (Digistat - Ascom Holding AG, Baar, Switzerland). Demographic information (gender, age), clinical information (fracture side, ASA score, medical diagnosis), peri and intra-operative data: time of surgery (day shift: 07:00–19:00, night shift 19:00–07:00), experience of the surgeon (resident vs. board certified), time to surgery or preoperative waiting time (defined as time from admission to incision), and complications (wound complications other than infection, delirium, infection (other; as urinary tract infection, pneumonia), PJI, PPF, acetabular erosion, dislocation) were extracted manually. Suspicion of infection was raised based on a combination of clinical indicators, including increased wound discharge, wound dehiscence, focal pain, and fever, alongside abnormal biological parameters such as elevated C-reactive protein (CRP) levels and leukocyte count. The primary endpoint was indication for surgical revision within 4 years from HA.

### Surgical intervention

**Fig. 1:**
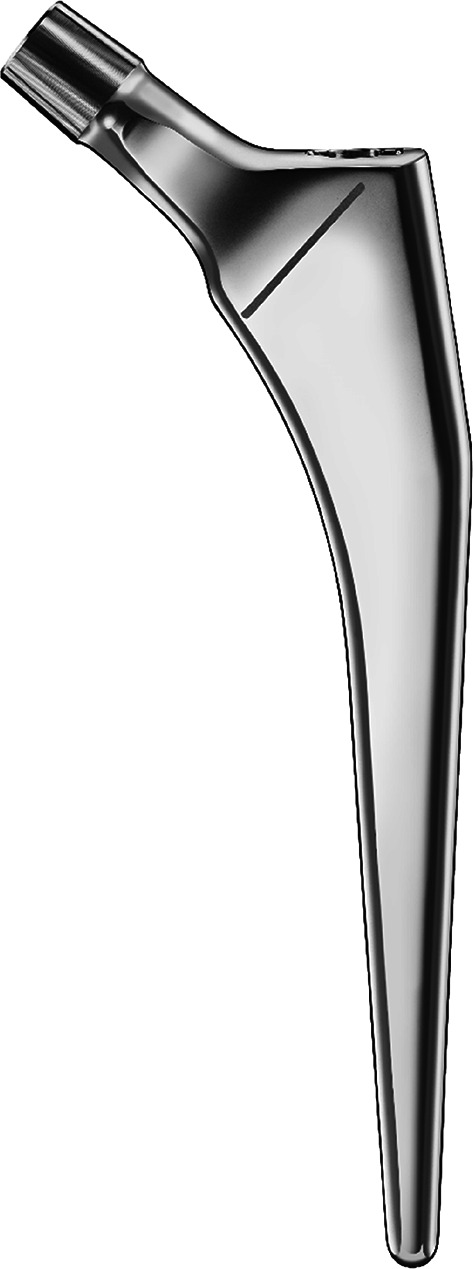
The HARMONY^®^ cemented stem (Image courtesy of Symbios Orthopédie SA; used with permission)

**Fig. 2:**
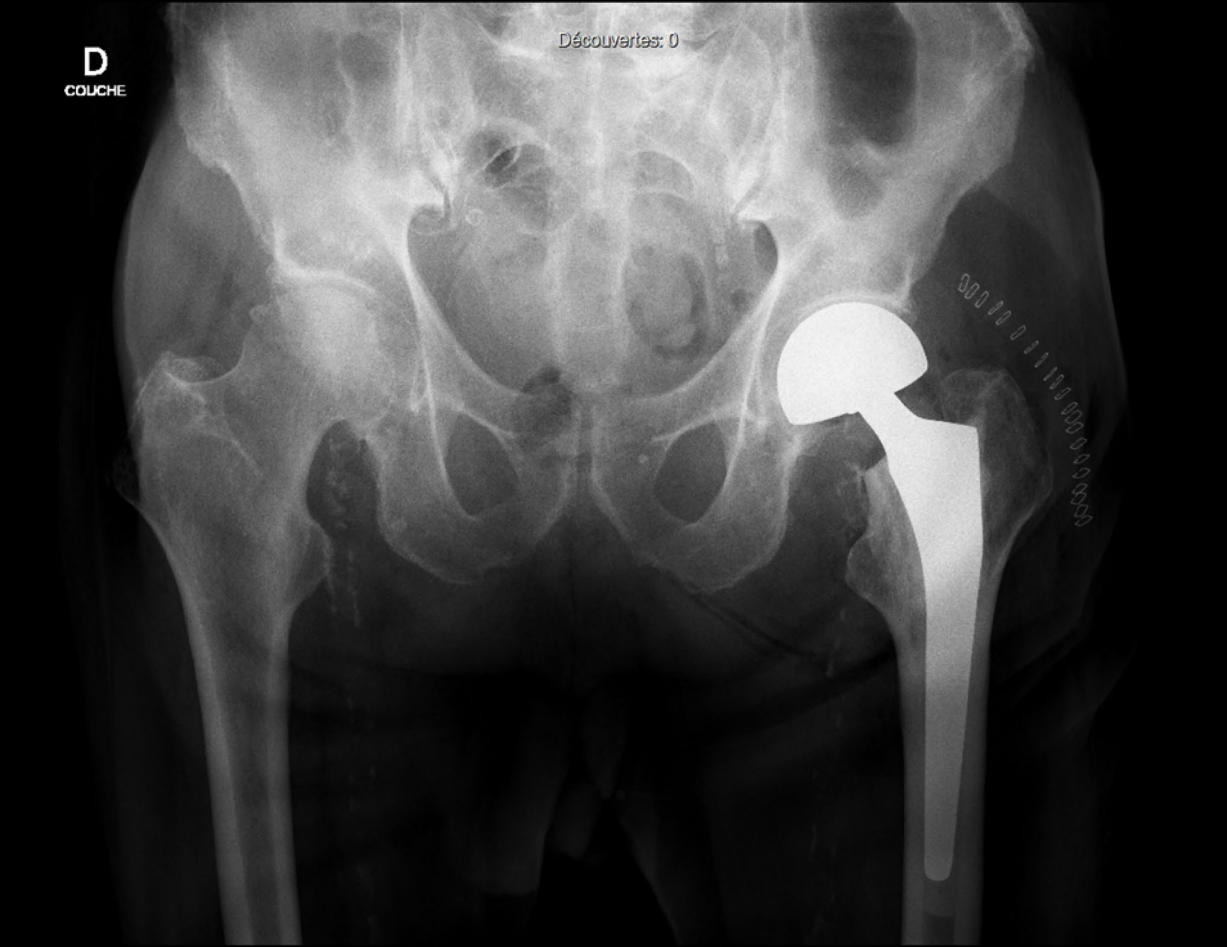
Postoperative anteroposterior pelvis radiography obtained within 24 h of surgery

All patients underwent surgery by a team of board-certified orthopedic surgeons and a resident surgeon. Procedures were performed under either general or spinal anesthesia, with a posterior approach (Moore) being the standard technique at our institution. A tapered, polished, stem (Harmony, Symbios Orthopédie S.A, Yverdon-les-Bains, Switzerland – Fig. 1), cemented via a 3rd generation classic technique coupled with a bipolar head (OHST, Medizintechnik, Rathenow, Germany) was used in the majority of patients. Postoperative radiography to confirm correct implant position was obtained within 24 h of surgery (Fig. 2) and patients were encouraged to early weight-bearing with a walker or two crutches, under the supervision of a specialized physiotherapist. Patient follow-up was conducted according to our protocol (6 weeks, 3 and 6 months, 1 year, and annually thereafter).

### Statistical analysis

We used SPSS version 26.0 (SPSS, Chicago, IL, USA) for data analyses. The Mann–Whitney U test was used to assess continuous variables, and chi^2^ or Fisher exact test for categorical variables. Clinically relevant non collinear covariates, assessed through variance inflation factor, were used in multivariable analysis. After checking Cox assumptions, a multivariable Cox proportional hazards regression models were performed with time-to-event set at 4 years. Adjusted hazard ratios (aHRs) and 95% confidence intervals (CIs) were calculated to evaluate the strength of any association. All statistic tests were 2-tailed and *P* < 0.05 was considered statistically significant. We created Kaplan-Meier curves of primary endpoint free survival stratified by sex, age, ASA score, and timing of the operation (day vs. night shifts).

## Results

**Fig. 3:**
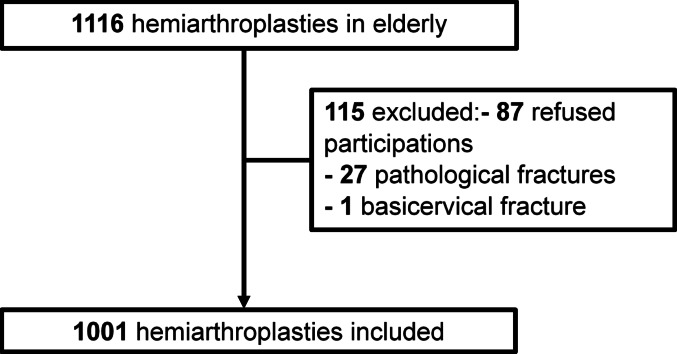
Study population

We included 1,001 patients (Fig. 3) with a median age of 86 years (interquartile range [IQR] 80–90) years and a median follow-up of 32 months (IQR 6–48 months). The patients were predominantly female (741; 74%), with two-thirds having multiple comorbidities (ASA ≥ 3). Most surgeries were conducted during the day shift (804, 80.3%), and half of them within 24 h of trauma (Table 1). 

**Table 1:** Baseline patient characteristics, operative data and post-operative non-hip related complications

	All patients (*n* = 1001)	No indication for revision surgery (*n* = 904)	Indication for revision surgery (*n* = 97)	*p*
Demographics				
Female sex	741 (74)	673 (74)	68 (70)	0.394
Age (years)	86 (80–90)	86 (81–91)	85 (79–87)	0.008*
ASA classification				
I	13 (1)	11 (1)	2 (2)	
II	344 (34)	311 (34)	33 (34)	
III	595 (59)	537 (59)	58 (60)	0.912#
IV	49 (5)	45 (5)	4 (4)	
Details about primary intervention				
Left femur	508 (51)	464 (51)	44 (45)	0.286
Timepoint				
Day shift	804 (80)	730 (81)	74 (76)	
Night shift	197 (20)	174 (19)	23 (24)	0.285
Operation within 24 h	563 (56)	513 (57)	50 (52)	0.334
Duration of operation				
≤90 min	474 (47)	422 (47)	52 (54)	
>90 min	527 (53)	482 (53)	45 (46)	0.201
Board certified surgeon	390 (39)	352 (39)	38 (39)	1.000
Non-hip related complications (within one month post operatively)				
Stroke	16 (2)	16 (2)	0 (0)	0.390
Septic shock	3 (0.3)	3 (0.3)	0 (0)	1.000
Mechanical fall	99 (10)	76 (8)	23 (24)	< 0.001*
Infection other than prosthetic joint infection	157 (16)	142 (16)	15 (16)	1.000
Delirium	131 (13)	116 (13)	15 (16)	0.432
Skin pressure ulcus	117 (12)	108 (12)	9 (9)	0.509

Data are depicted as number or median and interquartile range (age). In parenthesis percentage

*statistically significant (*p* < 0.05)

^a^comparison ASA III/IV versus I/II

Postoperative complications (non-hip related) within one month after HA occurred in over half of all patients (523, 52.2%). These included 157 (15.7%) infections other than PJI, 131 (13.1%) cases of delirium, 117 (11.7%) patients with skin pressure ulcer, 3 cases of septic shock (0.3%), and 16 strokes (1.6%). Ninety-nine (9.9%) patients presented at least one fall within one month after HA.

A total of 97 patients (9.7%) presented with at least one hip related complication, with four patients presenting two distinct complications: PPF occurred in 49 (4.9%) patients, hip prosthesis dislocation in 30 (2.9%), PJI suspicion in 17 (1.7%), acetabular erosion in 3 (0.3%), and wound complications in 38 (3.8%). Forty patients (3.9%) underwent HA revision surgery (rHA).

Among the 49 patients with PPF, 12 hips (1.2%) had a Vancouver A fracture, only one of these required surgical fixation. Plate osteosynthesis was carried out in 29 hips (2.9%) for Vancouver B1, B2, B3 and C fractures. Three Vancouver B1 fractures (0.3%) were managed conservatively. One Vancouver B1 fracture (0.1%) and four hips (0.4%) with Vancouver type B2 or B3 factures required fracture fixation with plate osteosynthesis and stem exchange.

Dislocation was found in 30 hips (2.9%), with 14 cases (1.4%) successfully treated by closed reduction. Open reduction without implant revision was carried out in 1 hip (0.1%). A Girdlestone procedure was carried out in 1 hip (0.1%) because of recurrent dislocation. Open reduction and change of the bipolar head were necessary in 7 patients (0.7%), either following dissociation between the bipolar cup and the prosthetic head after closed reduction or to increase the offset. In 7 hips (0.7%) revision surgery was performed with conversion to THA using a dual-mobility cup while retaining the existing stem.

Seventeen patients (1.7%) had a suspicion of acute periprosthetic joint infection (PJI) of which two (0.2%) had a previous history of closed reduction for dislocation. They underwent revision surgery: via change of mobile parts and implant retention (*n* = 16, 1.6%) and one undergoing a Girdlestone procedure (0.1%). Acute infection was confirmed in 11 cases (1.1%): Pathogens identified include: *Staphylococcus MSSA* and *MRSA*, *Enterococcus faecalis*, *Staphylococcus epidermidis*, *Serratia marcescens*, *Proteus mirabilis*, *Pseudomonas aeruginosa*, *Escherichia coli*, and *Staphylococcus hominis*. Patients received pathogen-specific antibiotic therapy for the duration of 12 weeks. In cases where no pathogen was identified, antibiotic therapy was discontinued following multidisciplinary discussion with infectious disease specialists. One patient declined, leading to the creation of an iatrogenic fistula. The infection was eradicated in the other patients using this protocol.

Three patients (0.3%) were completed to THA due to pain and radiographic acetabular erosion.

Dislocations and PJI typically occurred early at a median of 34 days (IQR 20.5–56) and 25 days (IQR 19.5–34) postoperatively, respectively, whereas PPF presented later, with a median of 5 months (IQR 5–26) and acetabular erosion at a median of 14 months postoperaively (12.3–34.7).

The occurrence of hip-related complications was not affected by the timing of surgery (day vs. night), the duration of surgery (> 90 min), or the surgeon’s experience (board certification) (Table 1).

**Fig. 4:**
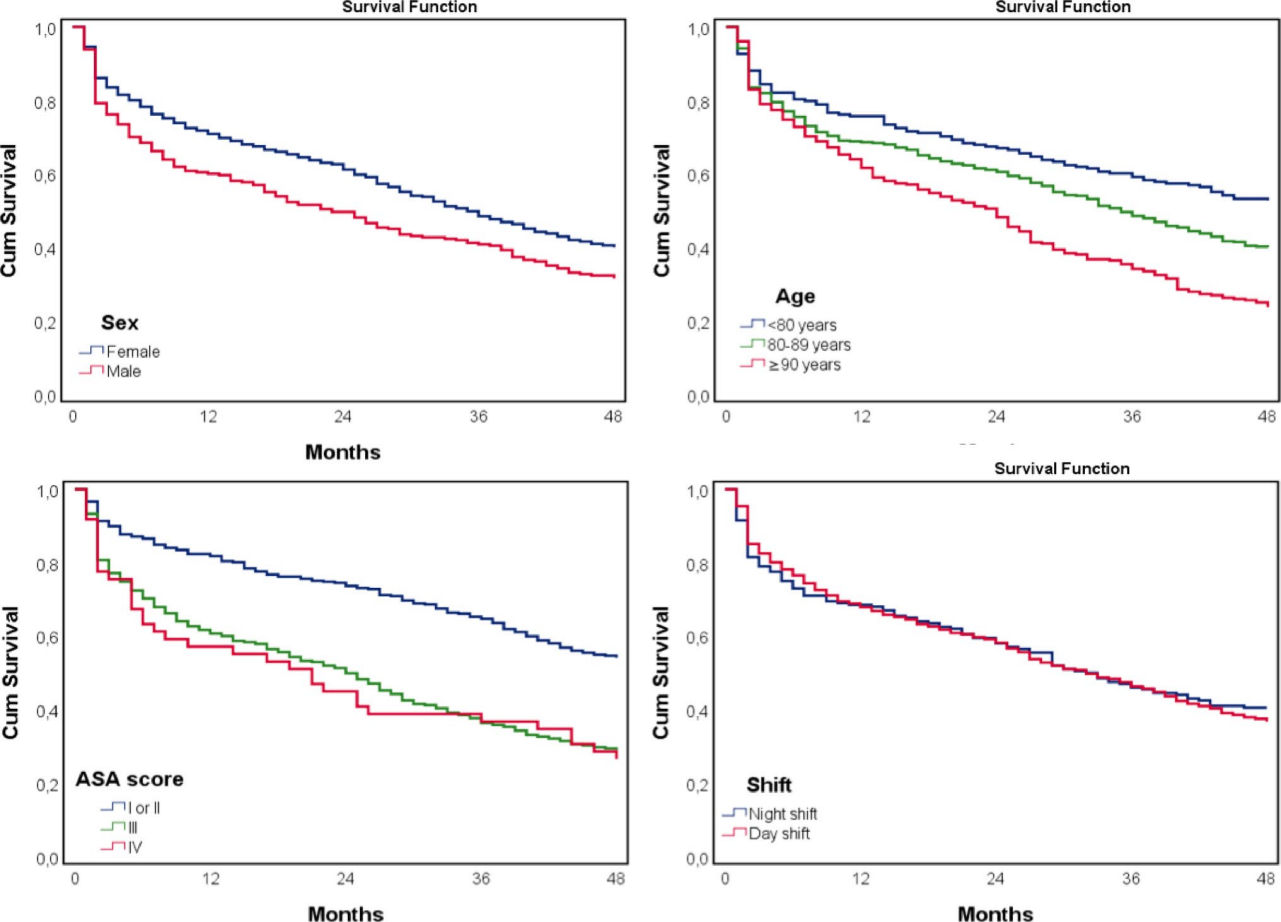
Kaplan-Meier curves showing probability of survival free of revision indication based on (A) sex, (B) age, (C) ASA score, and (D) timing of operation (night/day)

Cumulated mortality rates at 1 month, 6 months, 1 year and 4 years were 8%, 19%, 21%, and 51% respectively. Kaplan-Meier curves of primary endpoint free survival stratified by sex, age, ASA score, and timing of the operation (day vs. night shifts) are shown in Fig. 4. Table 2 depicts the comparison of patients who survived or did not survive within a 4-year postoperative period. In the Cox regression model, male sex (*P* = 0.008; aHR: 1.28, 95% CI 1.07–1.54), age > 80 years (*P* < 0.001; aHR: 1.86, 95% CI 1.50–2.31), ASA classification III or IV (*P* < 0.001; aHR: 2.11, 95% CI 1.74–2.55), and delirium within one month postoperatively (*P* = 0.030; aHR: 1.29, 95% CI 1.02–1.61) were associated with increased 4-year mortality (Table 3).

**Table 2:** Predictors of 4-year mortality

	Survivors (*n* = 416)	Non-survivors (*n* = 585)	*P*
Demographics			
Female sex	322 (77)	419 (72)	0.041
Age (years)	83 (78–88)	88 (83–92)	< 0.001
ASA classification			
I	12 (3)	1 (0.2)	
II	197 (47)	147 (25)	
III	191 (46)	404 (69)	< 0.001*
IV	16 (4)	33 (6)	
Primary operation on left femur	202 (49)	306 (52)	0.249
Operative data			
Day shift	326 (78)	478 (82)	
Night shift	90 (22)	107 (18)	0.197
Operation within 24 h	257 (62)	306 (52)	0.003
Duration of operation			
≤90 min	183 (44)	291 (50)	
>90 min	233 (56)	294 (50)	0.083
Board certification	172 (41)	218 (37)	0.212
Complications (within one month post operatively)			
Stroke	6 (1)	10 (2)	0.804
Septic shock	0 (0)	3 (0.3)	0.576
Mechanical fall	40 (10)	59 (10)	0.831
Infection other than prosthetic joint infection	54 (13)	103 (18)	0.052
Wound complications other than infection (hematoma, seroma, wound discharge or dehiscence)	19 (5)	19 (3)	0.315
Delirium	38 (9)	93 (16)	0.002
Cutaneous chronic lesion such as eschar or ulcer (not related to the operated femur)	48 (12)	69 (12)	0.921
Revision surgery (within 4 years from initial operation)	14 (3)	26 (4)	0.418

Data are depicted as number and percentage or median and interquartile range

*comparison ASA III/IV versus I/II

**Table 3:** Cox proportional hazards regression model of four-year mortality

	*P*	aHR (95% CI)
Male sex	0.008*	1.28 (1.07–1.54)
Age > 80 years old	< 0.001*	1.86 (1.50–2.31)
ASA classification III or IV	< 0.001*	2.11 (1.74–2.55)
Operation within 24 h	0.285	0.91 (0.77–1.08)
Delirium#	0.030*	1.29 (1.02–1.61)
Infection other than prosthetic joint infection#	0.226	1.15 (0.92–1.61)
Any indication for revision surgery	0.764	0.96 (0.72–1.27)

*aHR* adjusted hazard ratio, *CI* confidence interval

^a^within one month post operatively

*statistically significant (*p* < 0.05)

## Discussion

We found that cemented HA via a posterior approach was a safe and reliable treatment for FNF in elderly, delivering consistent low revision rates and complications. Avoiding rHA is primordial in this fragile patient group [26]. We were unable to identify independent risk factors for revision other than older age. Our population was highly comorbid as evidenced by ASA rates above III and a mortality rate of over 50% at 4 years.

Postoperative complications such as delirium have been commonly cited after FNF surgery (23–39%) and associated with high one year mortality rates [27, 28]. We did not demonstrate higher rates of rHA for patients with postoperative delirium.

Only 3.9% of patients had rHA, these rates are in line with the literature (3%−8%) [29–32]. In this study we report 3 cases (0.3%) of acetabular erosion requiring conversion to THA, which is similar to other studies including a large Australian based registry study of 21,717 bipolar HA [30, 33]. These results, along with those from the literature, suggest that the choice of THA should not be based on the potential risk of acetabular erosion, but rather on other criteria [24].

Our mortality rates highlight the fragility of the ever-aging geriatric population and were similar to a German population registry study of HA and other studies [31, 32, 34]. Cox regression analysis exhibited age above 80 years, delirium within one month and ASA classification of III + IV as independent risk factors for increased mortality at 4 years postoperatively (aHR 1.86, 1.29 and 2.11 respectively). These results are concordant with the literature [28, 34]. This highlights the necessity for conjoint patient management by specialized ortho-geriatric teams [27].

Falker et al. discovered that perioperative factors such as preoperative waiting time, early surgical complications, or experience of the surgeon were not associated with a higher overall mortality [34]. In accordance with other studies [35, 36], we did not find an increased indication for revision or mortality when non-board-certified surgeons operated.

Waiting times for surgery longer than 24 h for FNF are often considered undesirable due to an increased risk of complications and mortality [37]. This was not reflected in our study. Delayed surgery for medical optimization in comorbid patients may not affect mortality and could be beneficial [38, 39]. HA for FNF is often treated as an urgent procedure, leading to extended fasting periods and surgeries being scheduled at all hours, including night shifts, as daytime ORs are occupied by elective cases. A retrospective study by Chacko et al. of 767 patients (night vs. day surgery) found no significant difference in complications or mortality rates [40]. Although studies suggest potential cost reductions for night surgeries, this remains hypothetical and requires further prospective research. Our findings along with others also showed no association between time of surgery (night vs. day) and outcomes [41, 42]. These results confirm that HA for FNF can be scheduled electively, enabling preoperative preparation for comorbid patients, without increasing risks.

The PJI rates reported in our study were similar to those in the literature (2%−3%) [36, 43]. PPF rate was slightly higher than in the literature, with some studies citing as low as 0.9% [44–46]. This finding may be explained by the high comorbidity and heterogeneity of our patients. PPF occurred multiple months after return to home/nursing care which is concordant with the literature [44].

Dislocation occurred as an early postoperative complication with an incidence comparable to existing literature (0.8%−13%) [32, 47, 48]. Despite the high risk of recurrent dislocation following the first episode, current guidelines recommend closed reduction as the initial management strategy with rHA reserved for two or more dislocations [48, 49]. However, rHA with conventional single-bearing constructs does not guarantee stability, as dislocation rates remain high (up to 22% in some series) particularly when smaller femoral head diameters are used [50]. Dual-mobility (DM) constructs may confer greater stability. Chalmers et al. reported no postoperative dislocations at two years in revision HA to THA with DM constructs and compared this to large femoral heads [51].

Most randomized control trials or meta-analyses comparing THA to HA (bipolar or unipolar) don’t include DM bearings despite evidence of their benefit in reduction of dislocations in high risk populations in primary and revision settings [24, 52–54]. Certain studies support the use of DM THA in FNF treatment reporting low dislocation rates [55, 56]. A recent Swiss registry study found no significant difference in 10-year revision rates or implant survivorship between HA and DM THA for FNF in patients aged 65 to 84 years [57]. These findings underscore the need for individualized arthroplasty selection based on age, mobility and comorbidities.

The surgical approach for HA in comorbid patients at higher risk of dislocation remains a topic of debate as certain studies have demonstrated high dislocation risk via the posterior approach in patients with dementia [24, 58–61]. We demonstrate comparable dislocation rates to the literature. However, the absence of a control group limits the external validity of our study. Interestingly only 13.9% of all HA for fracture are conducted via the posterior approach according to SIRIS [2]. A multicenter, RCT involving 843 patients comparing the posterolateral approach and direct lateral approach for HA in FNF found no difference in secondary outcomes between the two groups. However, the posterolateral group exhibited a higher dislocation rate (5.5%) and increased revision surgery both higher than that observed in our cohort [62]. The strong point of this study lies in its inclusion of patients with dementia and cognitive impairment. The direct anterior approach for HA in FNF is also increasing in popularity with a potential for superior early functional mobility [63]. A recent metanalysis supports this, demonstrating a significantly higher rate of dislocation via the posterior approach without a significant increase in revision surgery or mortality between the two approaches [63]. Comparisons with the lateral approach found no early functional advantage of the anterior approach for HA therefore, its widespread adoption is not recommended [64]. These meta-analyses emphasize the heterogeneity of this patient population and the difficulty of creating generalizable treatment pathways.

This study is limited by its retrospective and single center design impacting external validity. Further, the lack of a control group, absence of clinical scores and radiographic analysis limits results and conclusions. The limited sample size may explain the lack of statistically significant data.

In conclusion, cemented HA via a posterior approach in our cohort was a safe and reliable treatment for FNF, with complication rates being comparable to the literature. Independent risk factors for increased mortality include age, ASA score III or IV and delirium. Timing of surgery (night/day) and board certification did not significantly affect revision rates; thus, HA for FNF in absence of another indication for emergent surgery, might be performed during day shifts, in dedicated trauma rooms, even after 24 h from FNF, potentially easing the strain on healthcare staff and facilities during night shifts. More prospective studies are needed to evaluate the type of surgical approach for FNF in the geriatric population.

## Data Availability

No datasets were generated or analysed during the current study.
